# Single-cell and pseudobulk analyses reveal hidden mitochondrial expression imbalance in gastric cancer

**DOI:** 10.3389/fgene.2026.1826214

**Published:** 2026-06-17

**Authors:** Le Chang, Jingwen Hou, Yingying Li, Simeng Liu, Pengya Feng, Ya Li, Chunjing Qiu, Pengyuan Zheng, Xia Xue, Yang Mi

**Affiliations:** 1 Henan Key Laboratory for Helicobacter pylori and Digestive Tract Microecology, The Fifth Affiliated Hospital of Zhengzhou University; Institute of Rehabilitation Medicine, Henan Academy of Innovations in Medical Science; Tianjian Laboratory of Advanced Biomedical Sciences, Zhengzhou University, Zhengzhou, Henan, China; 2 Henan Provincial Outstanding Overseas Scientists Chronic Liver Injury Workshop, Henan Joint International Research Laboratory of Chronic Liver Injury, Zhengzhou, Henan, China; 3 Department of Gastroenterology, Fifth Affiliated Hospital of Zhengzhou University, Zhengzhou, China

**Keywords:** gastric cancer, gene expression, mitochondrial homeostasis, precise medicine, single cell sequencing data

## Abstract

**Background:**

Mitochondrial function is essential for biology, particularly in cancer. However, cell-type-specific expression patterns of conserved mitochondrial genes in gastric cancer (GC) remain unclear. We herein raised and tested a novel hypothesis of “mitochondrial conserved gene expression homeostasis imbalance” in GC cohorts with single-cell resolution.

**Methods:**

This work analyzed an open-accessed scRNA-seq dataset (GSE206785, 24 GC patients, 48 samples) with Seurat and defined 43 clusters grouped into 15 cell subtypes. In parallel, Pseudobulk profiles were generated to simulate bulk RNA-seq. A mitochondrial conserved gene score was computed by Seurat AddModuleScore, GSVA, and AUCell. Mitochondria-related biomarkers were also screened, validated, and incorporated into a mitochondria-dependent prognostic model that was further evaluated.

**Results:**

Without considering cell-type-specific expression patterns, Pseudobulk analysis showed no significant differences in mitochondrial conserved gene expression between GC and control samples. In contracst, single-cell analysis found a cell-type-specific imbalance, under which tumor-associated epithelial cells displayed relatively elevated mitochondrial conserved gene expression, while non-epithelial cells showed reduced. Notably, survival analyses identified gene *KRT7* and *KLRC1* as robust prognostic biomarkers for early GC.

**Conclusion:**

Our findings support a mitochondrial conserved gene expression homeostasis imbalance in GC, which is characterized by compartment-specific mtGene expression imbalance. Also, *KRT7* and *KLRC1* emerge as prognostic markers for therapies aimed at restoring mitochondrial homeostasis in GC.

## 1 Introduction

Mitochondria are double-membrane organelles responsible for cellular energy production, calcium signaling, and apoptosis regulation, it maintains cellular metabolic homeostasis and energy supply (Harrington et al., 2023). As a relatively independent organelle due to owning genetic materials and self-replicing ability, mitochondria hold specific conserved mitochondrial DNA (mtDNA), which encodes 13 core subunits of the oxidative phosphorylation (OxPhos) system (Scarpulla, 2008). The mitochondrial genes were usually regarded as evolutionarily conserved with minor inter-tissue expression variation, generally following the rule of metabolic consistency across tissues (Barshad et al., 2018; Shtolz and Mishmar, 2023). However, recent studies have suggested that the expression of mitochondrial genes can vary among different tissues, especially cell types, due to diverse micro-environments, and this is particularly more pronounced at the single-cell level (Burr et al., 2023; Nitsch et al., 2024). The uneven expression of OxPhos-related genes is closely related to cell fate and disease progression, for instance, OxPhos defects lead to hypermetabolism and reduced lifespan in cells and patients with mitochondrial disorders (Sturm et al., 2023). In parallel, mitochondrial homeostasis imbalance drives tumor progression by promoting metastasis, chemo resistance, and immunosuppression under the tumor microenvironment (Zhang et al., 2022; Chen et al., 2024). The alternation of mitochondrial gene expression promotes the progression of oral squamous cell carcinoma, featured by EMT (epithelial mesenchymal transition)-driven metastasis and an immuno-suppressive microenvironment via M2 polarization (Kuo et al., 2020). It has been confirmed that tumors hijack mitochondrial functions through metabolic reprogramming, rewiring OxPhos, enhancing Reactive Oxygen Species (ROS) generation, and altering metabolic processes to fuel proliferation (Zhang et al., 2023). Understanding the interaction between mitochondrial homeostasis imbalance and tumors would pave the way for developing potential therapeutics of cancers targeting mitochondrial homeostasis from a metabolic reprogramming perspective.

Gastric cancer (GC) is a common type of malignant tumor in the digestive system with both a high incidence rate and mortality rate, posing a major global health burden to human beings (Bray et al., 2024). It has been found strongly associated with risk factors including *Helicobacter pylori* infection, dietary habits (e.g., high intake of smoked, salted, or processed foods; low vegetable/fruit consumption), tobacco smoking, obesity, and genetic syndromes (e.g., hereditary diffuse gastric cancer, Lynch syndrome) (Wroblewski and Peek, 2010). Current clinical management strategies for GC mainly are endoscopic submucosal dissection (ESD), traditional surgeries, neoadjuvant/adjuvant chemotherapy/radiotherapy, targeted therapy, and immunotherapy. While early diagnosis significantly improves the prognosis of GC through treatment such as ESD, effective therapeutic options for advanced stages are somewhat limited. The 5-year survival rate for advanced GC remains below 30% (Smyth et al., 2020; Guan et al., 2023), reminding the critical imperative to discover new targets and more potent treatment strategies. Recent studies have suggested that the occurrence and development of GC are closely related to the reprogramming of tumor metabolism (Liu et al., 2019). Previous studies have revealed that tumor cells with defective OxPhos function have stronger invasive and drug-resistant potential, as well as mitochondrial dysfunction can promote tumor progression by inhibiting the activity of immune cells in GC (Zhao et al., 2022). The single-cell studies reveal that GC tumor microenvironment heterogeneity, particularly in mitochondrial metabolic reprogramming, may underpin treatment failure, motivating our investigation into cell-subtype-specific mitochondrial dysregulation (Wang et al., 2023). However, given the traditional standpoint that mitochondria are evolutionarily conserved, the role of mitochondrial gene expression homeostasis in GC is limited discussed, especially since the expression differences at the cellular sub-population levels are probably masked by bulk sequencing.

This study aims to reveal the expression characteristic dynamics of mitochondrial conserved genes (*ATP6, ATP8, COX1, COX2, COX3, CYTB, ND1, ND2, ND3, ND4, ND4L, ND5,* and *ND6*) in 15 different cell populations and summarize the cell-type-specific mitochondrial conserved gene expression patterns. We first proposed the “mitochondrial conserved gene expression homeostasis imbalance hypothesis”, which refers to although conserved mitochondrial gene expression is relatively stable in evolution, the intercellular heterogeneity of their expression reflects the disruption of homeostasis. This imbalance may promote GC tumor occurrence and progression through mechanisms including regulating metabolic pathways, oxidative stress, and immune evasion. Furthermore, we screened mitochondria-related prognostic biomarkers and established a mitochondria-dependent prognostic model in GC, providing a theoretical foundation for understanding mitochondrial homeostasis imbalance in GC diagnosis and treatments.

## 2 Materials and methods

### 2.1 Workflow design

**FIGURE 1:**
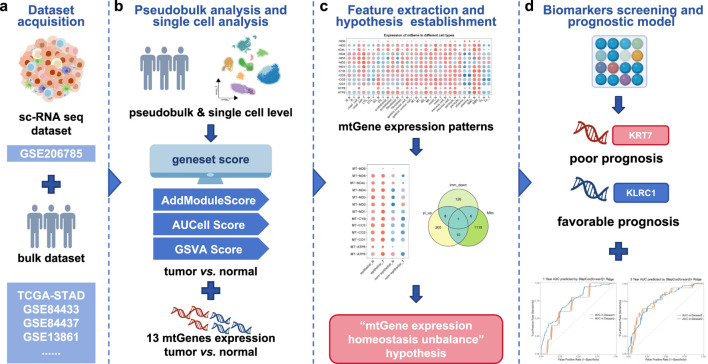
Workflow of the research. **(a)** Dataset acquisition: Collection and annotation of GC single-cell transcriptomic data (GSE206785) and multiple bulk transcriptomic datasets (e.g., TCGA-STAD, GSE84433, GSE84337, GSE13861). **(b)** Pseudobulk analysis: Simulation of bulk RNA-seq characteristics by merging single-cell data to evaluate expression differences of 13 mtGenes between tumor and normal tissues at the gene and gene set scoring levels; single-cell resolution analysis: heterogeneous expression of 13 mtGenes and their gene set scores across tumor-normal derived cell subtypes. **(c)** Feature extraction and hypothesis establishment: Summarize mtGene expression patterns across tumor and normal samples, and propose the hypothesis of “mtGene expression homeostasis unbalance”. **(d)** Biomarker screening and prognostic model construction: Identification of prognostic biomarkers (e.g., KRT7 and KLRC1) and establishment of mitochondria related GC prognosis model. GC: gastric cancer; GSVA: gene set variation analysis; mtGene: mitochondria gene; sc-RNA seq: single cell RNA sequencing.

This study explored the imbalance of mitochondrial gene expression homeostasis in GC with four phases. The framework of the experimental design, including key methodological steps and analytical strategies employed at each phase, was provided in Figure 1.

### 2.2 Data obtain and processing

The single-cell RNA sequencing (scRNA-seq) dataset of GC was obtained from a public platform. Using the search terms: ((((gastric cancer) OR (GC) OR (gastric tumor) OR (stomach cancer)))) AND (((single-cell RNA sequencing) OR (scRNA-seq))). We obtained the GSE206785 dataset (Illumina HiSeq 4000 platform, *Homo sapiens*, high-throughput sequencing) from the GEO.

To perform survival analysis for underlying marker genes, we downloaded and annotated several GEO datasets, including GSE84433 (Illumina HumanHT-12 V3.0 expression beadchip, *Homo sapiens*, array, *n*=357), and GSE84437 (Illumina HumanHT-12 V3.0 expression beadchip, *Homo sapiens*, array, *n*=433). To conduct a prognostic model, the TCGA-STAD dataset (RNA-seq, *Homo sapiens*, n=379) was acquired from TCGA as training cohorts, and GSE18361 (Illumina HumanWG-6 v3.0 expression beadchip, *Homo sapiens*, array, *n* = 65) was processed as validation cohorts.

### 2.3 scRNA-seq data analysis

ScRNA-seq analysis was conducted by using the *Seurat* (v4.4.0) R package (Hao et al., 2021). The raw data from 111,140 cells underwent standardized quality control, including doublet removal and filtering criteria (retained cells: nFeature RNA 200-5,000; nCount RNA 500-50,000; mitochondrial gene ratio ≤10%), resulting in 98,124 high-quality cells for further analysis. The raw count matrix was normalized by library size and scaled via log-normalization. Batch effects across samples were corrected using the *Harmony* R package (v1.1.0). Highly variable genes were identified using the FindVariableFeatures function. Principal component analysis (PCA) was performed for linear dimensionality reduction with default parameters. Cell clustering was implemented through nearest neighbor graph construction and shared nearest neighbor (SNN) modularity optimization (dims=21, resolution=1.5). Uniform manifold approximation and projection (UMAP) was subsequently applied using the RunUMAP function for visualization. The 43 distinct clusters were identified with cluster-specific upregulated markers detected via the FindAllMarkers function and classical cell markers were used to infer cell types (Zh et al., 2019).

### 2.4 Multidimensional cell atlas visualization with clusterGVis

To comprehensively analyze cellular heterogeneity in the single-cell transcriptomic data, multidimensional visualization was performed using the *clusterGVis* R package (v0.1.1). Gene Ontology (GO) enrichment analysis (Gene Ontology Consortium (2015)) was performed to annotate the biological functions of cell clusters using the *clusterProfiler* R package (v4.10.0) (Yu et al., 2012).

### 2.5 Pseudobulk analysis

To investigate population-level mitochondrial gene expression patterns in single-cell data, pseudobulk analysis was performed on the GSE206785 dataset, which includes paired tumor-normal tissue scRNA-seq profiles from 24 GC patients. This approach reduced the inherent sparsity bias of single-cell data by simulating a bulk RNA-seq quantification pattern, while preserving biologically meaningful inter-individual variability. The workflow included two stages: (1) Integration of raw single-cell count matrices by tissue-specific sample IDs to generate pseudobulk count matrices for tumor and normal tissues of each patient, (2) Library size normalization followed by calculation of relative expression abundance across all samples. This approach enables cross-sample comparability of gene expression levels by combining single-cell resolution with bulk-level robustness.

### 2.6 Mitochondrial conserved gene set score

To evaluate the gene set activity of 13 mitochondrial conserved genes (*ATP6, ATP8, COX1, COX2, COX3, CYTB, ND1, ND2, ND3, ND4, ND4L, ND5,* and *ND6*), three mainstream and complementary scoring algorithms were applied:

1. AddModuleScore in *Seurat* R package (v4.4.0) (Hao et al., 2021), calculating background-corrected scores by subtracting 100 random control gene sets matched to the target expression distribution.
2. GSVA score in *GSVA* R package (v1.48.3) (Ha et al., 2013), performing non-parametric enrichment analysis *via* Kolmogorov-Smirnov test on log(CPM+1)-normalized matrices.
3. AUC score in the *AUCell* R package (v1.50.0), computing Area Under the Curve (AUC) scores through gene ranking with dynamic thresholds (k=5%-20% of total genes).

For pseudobulk comparisons, single cell mtGene scores were further aggregated according to tissue-specific sample identifiers to generate pseudobulk score profiles for analyses.

### 2.7 Identification of mitochondria-related prognostic biomarkers

To identify potential mitochondria-associated markers, we compiled a comprehensive mitochondrial gene list by merging genes from MitoCarta3.0 and GeneCards. This list was intersected with DEGs identified from tumor epithelial cells and immune-downregulated populations to extract candidate prognostic biomarkers, respectively. Candidate genes were further validated in bulk datasets to evaluate their consistency in expression trends and prognostic associations.

### 2.8 Prognostic model construction and validation

The *Mime* R package (v1.0.0) was used to construct a prognostic model integrating mitochondria-related gene signatures (Liu et al., 2024). Model performance was assessed by concordance index (C-index) and time-dependent ROC analyses at 1-, 3-, and 5-year. Patients were stratified into high- and low-risk groups based on median risk score, and differences in overall survival were compared *via* Kaplan-Meier and log-rank tests.

### 2.9 Survival analysis

Survival analyses were performed on several GEO datasets (GSE84433, n=357; GSE84437, n=357; GSE84433, n=433; GSE18361, n=65; TCGA-STAD, n=379). Overall survival (OS) was defined as the time from diagnosis to death or last follow-up. Kaplan-Meier curves were generated using the survminer R package (v0.4.9), with patients stratified into high/low expression groups by median values of target genes or risk score. Inter-group differences were assessed *via* the log-rank test using the *survival* R package (v3.5.7).

### 2.10 Statistical analysis

All data cleaning, statistical analyses, and visualizations were performed using the R programme (version 4.3.1). Categorical variables were presented as counts (frequencies) and compared using the chi-square test. For normally distributed continuous variables, Student’s t-test (two groups) or one-way analysis of variance (≥3 groups) was applied. Non-normally distributed variables were analyzed using the Mann-Whitney test (two groups) or the Kruskal-Wallis test (≥3 groups). Post-hoc pairwise comparisons for Kruskal-Wallis results were adjusted via the Dunn-Bonferroni method. All statistical tests were two-sided, and a *P* value of less than 0.05 and a false discovery rate of less than 0.05 was considered statistically significant.

## 3 Results

### 3.1 Depict of the single cell landscape in GC

To investigate the cellular heterogeneity of GC at single-cell resolution, we processed a scRNA-seq dataset (GSE206785) comprising 48 samples (24 tumor tissues and paired non-tumor tissues) from 24 GC patients. After standardized quality control, a total of 98,124 cells were obtained from 24 GC and paired non-tumor samples, which were incorporated into further analysis. In the following gene expression normalization, we carried out PCA to reduce dimensionality and clustered cells based on the informative PCA space. UMAP visualizations were generated before and after harmony integration, revealing substantial mixing of cells across batches following correction while preserving the original clustering structure. To further quantify batch removal performance, we applied Local Inverse Simpson’s Index (LISI) analysis, which demonstrated a significant increase in batch mixing scores without compromising cell-type identity (Supplementary Figure S1).

**FIGURE 2:**
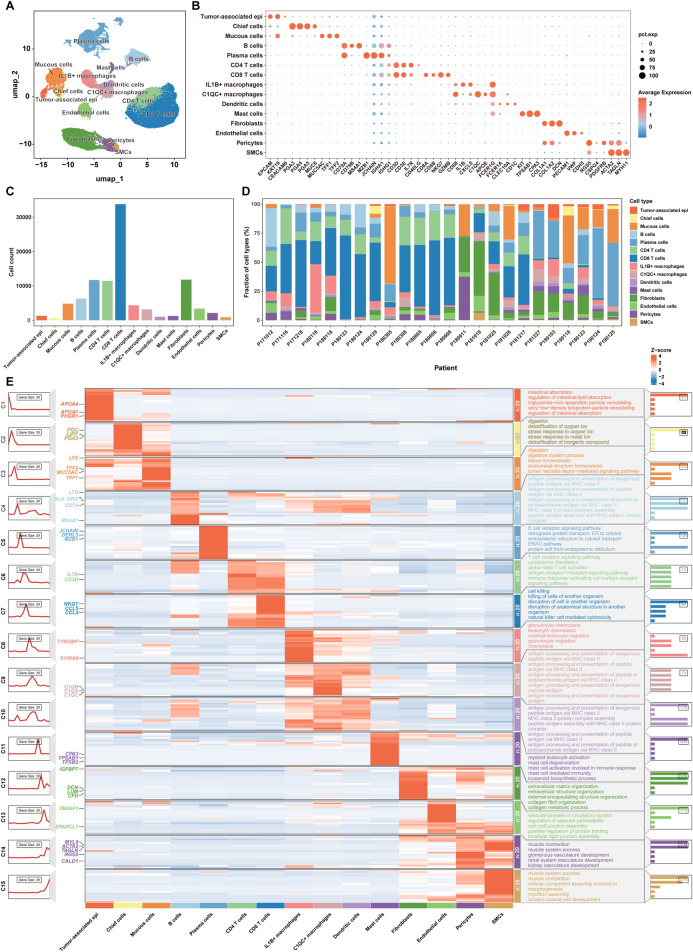
Single-cell transcriptomic landscape of gastric cancer. **(A)** UMAP visualization of the GSE206785 single-cell dataset showing cellular heterogeneity, with 43 clusters annotated into 15 major cell types. **(B)** Dot plot showing the expression patterns of canonical marker genes across different cell types. **(C)** Bar plot quantifying the absolute abundance of each cell subtype. **(D)** Stacked bar plot showing the relative composition of cell types across different patient sources. **(E)** Integrated visualization of cell clustering, representative marker gene expression, and GO functional enrichment analysis for annotated cell populations. UMAP: uniform manifold approximation and projection; GO: gene ontology.

The 43 cell clusters were identified based on differentially expressed genes (DEGs). Hierarchical cell annotation was subsequently performed, and cells were initially classified into three major lineages using canonical markers, including immune cells (*PTPRC*), stromal cells (*COL1A1, DCN*, *PECAM1*, et al.), and epithelial cells (*EPCAM*). Based on lineage-associated marker genes, these clusters were further annotated into 15 major cell subtypes (Figures 2A, B), including immune populations: IL1B+ macrophages (*CD68*, *IL1B*, *CXCL8*), C1QC+ macrophages (*C1QC*, *APOE*, *FCER1G*), dendritic cells (*FCER1A*, *CLEC10A*, *CD1C*), B cells (*CD79A*, *CD79B*, *MS4A1*), plasma cells (*MZB1*, *JCHAIN*, *IGHA1*, *IGHG1*), CD4 T cells (*CD3D*, *CD3E*, *IL7R*, *CD40LG*), CD8 T cells (*CD8A*, *CD8B*, *NKG7*, *GZMB*), and mast cells (*KIT*, *TPSAB1*, *CPA3*), stromal populations: fibroblasts (*COL1A1*, *COL1A2*, *DCN*), endothelial cells (*PECAM1*, *VWF*, *CDH5*), pericytes (*RGS5*, *CSPG4*, *PDGFRB*), and smooth muscle cells (*ACTA2*, *TAGLN*, *MYH11*); as well as epithelial populations, including mucous cells (*MUC5AC*, *TFF1*, *TFF2*), chief cells (*PGA3*, *PGA4*, *PGA5*), and tumor-associated epithelial cells *(EPCAM*, *KRT19*, *CEACAM6*). We quantified the cellular composition across all patients by calculating both absolute cell counts and relative proportions of each annotated cell subtype (Figures 2C, D). The single-cell transcriptional landscape was comprehensively visualized through several perspectives, such as lineage-defining marker activation patterns, subtype-specific marker expression, and functional ontology enrichment (Figure 2E).

In parallel, to exclude mitochondrial gene percentage (mtDNA%) bias, we performed complementary validation analyses. The mitochondrial conserved gene scores (mtScore) were calculated using multiple independent methods, including AddModuleScore, AUCell, and GSVA, all of which yielded highly consistent patterns, indicating that the observed signal is not dependent on a specific scoring approach. Notably, mtScore showed only a moderate correlation with mitochondrial gene percentage (mtDNA%), suggesting that it is not simply driven by mitochondrial read abundance. To further exclude the possibility that increased mtScore reflects cellular stress or apoptosis, we computed apoptosis-related gene scores and found no significant correlation with mtScore, indicating that the signal is unlikely to arise from damaged or dying cells. In addition, correlation analyses between mtScore and nuclear-encoded mitochondrial gene sets revealed weak or negligible associations, supporting the notion that mtScore captures a distinct transcriptional program rather than general mitochondrial content. All analyses were repeated under different quality control thresholds (mtDNA%<5% and <10%), and the results remained highly consistent, confirming the robustness of our findings (Supplementary Figures S2, S3).

### 3.2 Pseudobulk analysis of mitochondrial conserved gene expression in GC

**FIGURE 3:**
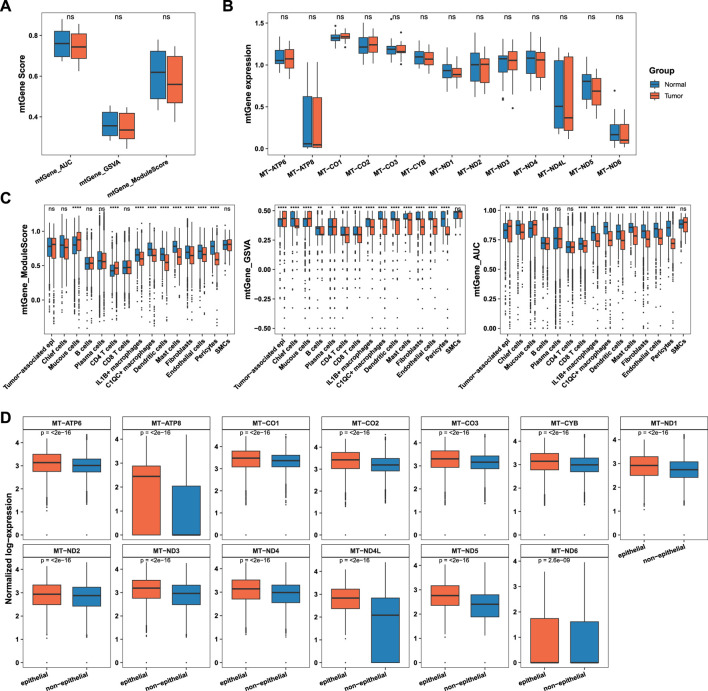
Compartment-specific mitochondrial gene expression patterns in gastric cancer. **(A)** Box plots showing pseudobulk-level mtGene gene-set scores (AUCell, GSVA, and AddModuleScore) between tumor and normal gastric tissues based on sample-level pseudobulk aggregation; boxes represent the interquartile range, and center lines indicate medians. **(B)** Box plots showing pseudobulk expression levels of 13 mtGenes between tumor and normal tissues. **(C)** Box plots comparing mtGene gene set scores across tumor-derived and normal-derived cell subtypes at the single-cell level; statistical significance is indicated (*p < 0.05, **p < 0.01, ***p < 0.001). **(D)** Box plots comparing normalized mtGene expression levels between epithelial and non-epithelial cellular compartments. GC: gastric cancer; GSVA: gene set variation analysis; mtGenes: mitochondria genes.

We conducted AddModuleScore, GSVA, and AUCell as gene set score methodologies to represent the overall situation of 13 mitochondrial conserved genes. These scoring algorithms were first applied at the single-cell level using normalized expression matrices, after which cell-level scores were aggregated according to tissue-specific sample identities to generate pseudobulk score profiles for paired tumor-normal comparisons. The score of 13 mitochondrial conserved genes calculated from 3 scoring systems all showed no significant difference between tumor and paired normal tissues (*P*>0.05, Figure 3A). Similarly, the expression of 13 mitochondrial conserved genes respectively have no significant difference between tumor and paired normal tissues (*P*>0.05), which further revealed conserved mitochondria expression patterns at bulk-level resolution (Figure 3B).

### 3.3 Single cell resolution profiling of mitochondrial conserved gene expression in GC

Intercellular variation overlapped and inter-patient variability emerged as the dominant source of heterogeneity, which may cover up tumor-normal differences in mitochondrial gene expression from a bulk-level perspective. Thus, we determined to explore mitochondrial conserved gene expression dynamics at single cell resolution. The score of mitochondrial conserved genes through three scoring systems in most specific-cell-subtype have significant difference between tumor and paired normal tissues in Figure 3C (*P*<0.05). The expression of most mitochondrial conserved genes in specific cell subtype performed significant difference between tumor and paired normal tissues (*P*<0.05, Supplementary Figure S4), which suggested suggesting compartment-specific mitochondrial transcriptional heterogeneity at single-cell resolution. These findings support that pseudobulk analyses covered up tumor-normal differences in conserved mitochondrial gene expression, and single-cell resolution analyses revealed significant cell subtype-specific mitochondrial transcriptional heterogeneity. In other words, the expression of conserved mitochondrial genes undergoes tumor microenvironment-dependent remodeling, can be detected at single-cell resolution. Similar results were observed under a more stringent mitochondrial gene filtering threshold (mtDNA%<5%, Supplementary Figure S5), indicating the robustness of these findings.

### 3.4 Cell-type-specific heterogeneity in mitochondrial conserved gene expression patterns in GC

To better understand the expression dynamics of mitochondrial conserved genes (mtGenes) in GC, we analyzed their expression patterns across tumor and normal tissues, as well as among different cell subtypes. We then investigated mtGene expression across various cell types derived from different tissue origins, which suggested that the differential expression patterns among similar cell types tended to be consistent, for instance, endothelial cells showed expression patterns similar to fibroblasts (Supplementary Figure S4). In most cases, the direction of mtGene expression change remained consistent across different cell types, although the magnitude of variation varied. To further generalize these observations, we compared mtGene expression between epithelial and non-epithelial compartments (Figure 3D). Epithelial compartments exhibited relatively higher mtGene expression levels, while non-epithelial compartments displayed relatively lower mtGene expression patterns, suggesting compartment-specific mitochondrial transcriptional imbalance in the gastric cancer microenvironment.

### 3.5 Exploration of mitochondria related genes in GC

**FIGURE 4:**
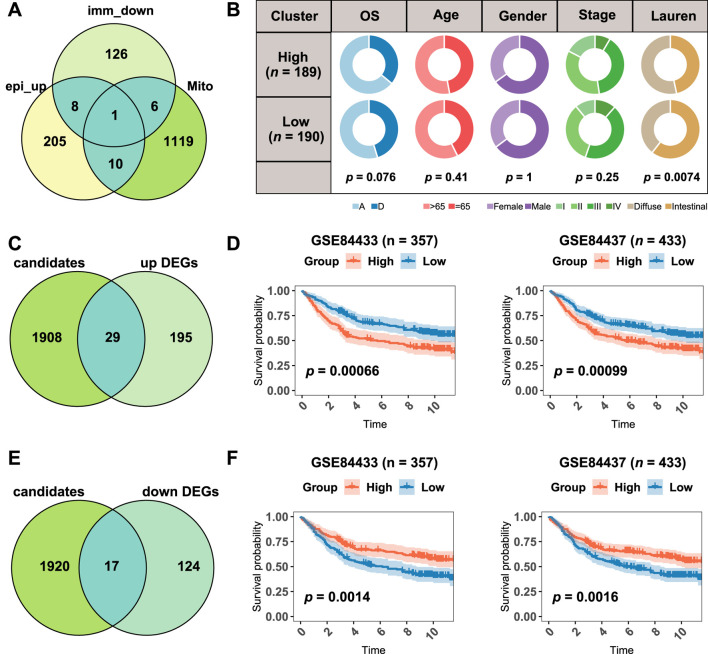
Identification and prognostic evaluation of mitochondria-related genes in gastric cancer. **(A)** Venn diagram showing the overlap among genes upregulated in tumor epithelial cells (epi_up), genes downregulated in tumor-infiltrating immune cells (imm_down), and mitochondria-related genes (Mito) from the MitoCarta3.0 database. **(B)** Distribution of clinical characteristics across high and low expression groups of MT-ND6 in the TCGA-STAD cohort; pie charts represent the proportion of samples in each category. **(C)** Venn diagram showing the intersection between mitochondria-associated candidate genes and upregulated differentially expressed genes (DEGs) in malignant epithelial cells. **(D)** Kaplan–Meier survival curves comparing overall survival between high and low KRT7 expression groups in GSE84433 and GSE84437 cohorts; shaded areas represent 95% confidence intervals. **(E)** Venn diagram showing the intersection between mitochondria-associated candidate genes and downregulated DEGs in tumor-derived immune cells **(F)** Kaplan–Meier survival curves comparing overall survival between high and low KLRC1 expression groups in GSE84433 and GSE84437 cohorts; shaded areas represent 95% confidence intervals. GC: gastric cancer; DEGs: differentially expressed genes; OS: overall survival.

The variation of mitochondrial conserved gene expression may be overlapped at the bulk level in GC, but the variation of the expression in mitochondria-related genes may be easily noticeable and detected. Here, mtGenes specifically refer to the 13 conserved mtDNA-encoded genes, whereas mitochondria-related genes additionally include nuclear-encoded genes associated with mitochondrial biology. Considering our hypothesis, we explored the mitochondrial related genes that are highly associated with metabolism in GC. Candidate mitochondrial-related genes were selected from the MitoCarta3.0 database (Supplementary Table S1, sheet 1). DEGs were determined from single-cell transcriptomic data. We identified 224 genes upregulated in tumor epithelial cells compared to normal epithelial cells (Supplementary Table S1, sheet 2), and 141 genes downregulated in tumor-infiltrating immune cells relative to their normal immune counterparts (Supplementary Table S1, sheet 3). The intersection of these two DEG sets had nine overlapping genes (*GSTP1, DDIT4, PPDPF, MT-ND6, NME2, ZFP36L2, EEF1G, RRBP1,* and *IGKC*), which were intersected with candidate genes. Only one gene, MT-ND6, was found to be mitochondria-associated (Figure 4A).

To further characterize the clinical significance of MT-ND6, patients from TCGA-STAD were divided into high and low ND6 expression groups. Chi-square analysis revealed no significant differences in survival status, age, sex, or stage between the two groups (Figure 4B). A significant association was observed with Lauren classification, where patients with diffuse-type GC were more frequently represented in the high-ND6 expression group.

### 3.6 KRT7 as a biomarker related to poor prognosis in GC

Mitochondrial conserved genes were identified as transcriptional indicators associated with compartment-specific mitochondrial expression patterns at the single-cell level. However, due to their limited detectability and susceptibility to cell composition bias in bulk transcriptomic data, these genes are not well suited for direct clinical application. To address this limitation, we further sought to identify surrogate biomarkers that are robustly detectable in bulk datasets while faithfully reflecting mitochondrial-related biological states.

Mitochondria-related genes have been associated with the development and progression of GC and have the potential to be a biomarker related to prognosis. To identify mitochondrial biomarkers associated with prognosis in GC, we expanded the MitoCarta gene set by integrating it with mitochondrial-related genes retrieved from the GeneCard database, thereby generating a more comprehensive list of mitochondria-associated candidates (Supplementary Table S1, sheet 4). We then intersected this gene set with the genes upregulated in malignant epithelial cells (Figure 4C), yielding biologically relevant mitochondrial-related candidates. We also performed survival analyses on mitochondria-related genes in several GC cohorts. Among 29 candidate genes, *KRT7* emerged as a stable predictor of adverse clinical outcomes. In the discovery cohort GSE84433 (n=357), increased *KRT7* expression correlated with reduced overall survival (*P*<0.05). This association was validated in independent cohorts (GSE84437, n=433, *P*<0.05, Figure 4D). Several additional cohorts showed no significant statistical differences, but they showed similar trends (Supplementary Figure S6, panel A). Therefore, *KRT7* can be regarded as a robust biomarker of poor prognosis in GC.

### 3.7 KLRC1 as a biomarker related to favorable prognosis in GC

We intersected the expanded mitochondria-related gene set with the genes downregulated in immune cells derived from tumor tissues to identify potential biomarkers associated with better prognosis (Figure 4E). We found *KLRC1* as a biomarker of favorable prognosis in GC. Survival analyses in the discovery cohort GSE84433 (n=357) revealed that high expression of *KLRC1* was associated with a favorable prognosis in GC (*P*<0.05). To verify its stability as a biomarker, survival analyses in another cohort were conducted: GSE84437 (n=433, *P*<0.05, Figure 4F). Several additional cohorts with no significant statistical differences showed similar trends (Supplementary Figure S6B). Therefore, the connection between *KLRC1* expression and overall survival supports its role as a biomarker of GC prognosis.

Under this framework, *KRT7* and *KLRC1* were identified as clinically translatable markers derived from mitochondrial-related gene selection strategies.

### 3.8 Hypothesis on compartment-specific mitochondrial gene expression heterogeneity in GC

**FIGURE 5:**
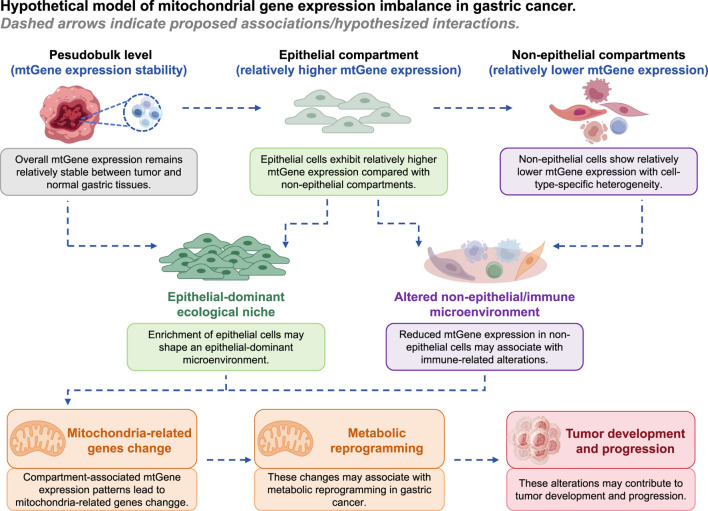
Hypothetical model of compartment-specific mitochondrial gene expression imbalance in gastric cancer. The schematic summarizes the proposed associations between compartment-specific mtGene expression patterns and gastric cancer progression. At the pseudobulk level, overall mtGene expression remains relatively stable between tumor and normal gastric tissues. However, single-cell analysis reveals relatively higher mtGene expression in epithelial compartments and relatively lower mtGene expression in non-epithelial compartments with cell-type-specific heterogeneity. These compartment-associated mtGene expression patterns may associate with epithelial-dominant ecological niches, altered non-epithelial/immune microenvironments, mitochondria-related transcriptional alterations, and metabolic reprogramming, potentially contributing to gastric cancer development and progression. Dashed arrows indicate proposed associations or hypothesized interactions rather than experimentally validated mechanisms. mtGene: mitochondrial gene.

Contrary to the prevailing view that mtDNA-encoded gene expression is globally conserved, our analyses revealed compartment- and cell-type-associated heterogeneity of mitochondrial gene expression in GC. Pseudobulk transcriptomic comparisons between tumor and adjacent normal tissues did not reveal significant differences in mtDNA-encoded gene expression. However, single-cell-resolution analyses uncovered lineage-associated transcriptional variation, in which tumor-associated epithelial compartments showed relatively higher expression of conserved mitochondrial genes, while non-epithelial (stromal and immune) compartments displayed relatively lower mtGene expression patterns. These compartment-associated transcriptional differences may reflect distinct metabolic demands and microenvironment-associated states across cellular populations within the GC microenvironment. These findings support a hypothetical model in which compartment-specific mtGene expression heterogeneity is potentially associated with gastric cancer progression (Figure 5).

### 3.9 Construction and validation of mitochondrial-associated prognostic model in GC

Based on the biological significance of mtGenes identified in our previous analyses, we constructed a mitochondrial-dependent prognostic model for GC using the *mime* machine learning R package. None of the 13 mitochondrial conserved genes achieved statistical significance univariate Cox regression analysis to screen survival-associated genes before model construction. Therefore, these genes were not retained in the final prognostic model, which was instead established based on survival-relevant features derived from bulk transcriptomic data. This result further supports the notion that, while mitochondrial conserved genes are informative for mechanistic characterization at the single-cell level, they may have limited direct prognostic value in bulk clinical datasets.

**FIGURE 6:**
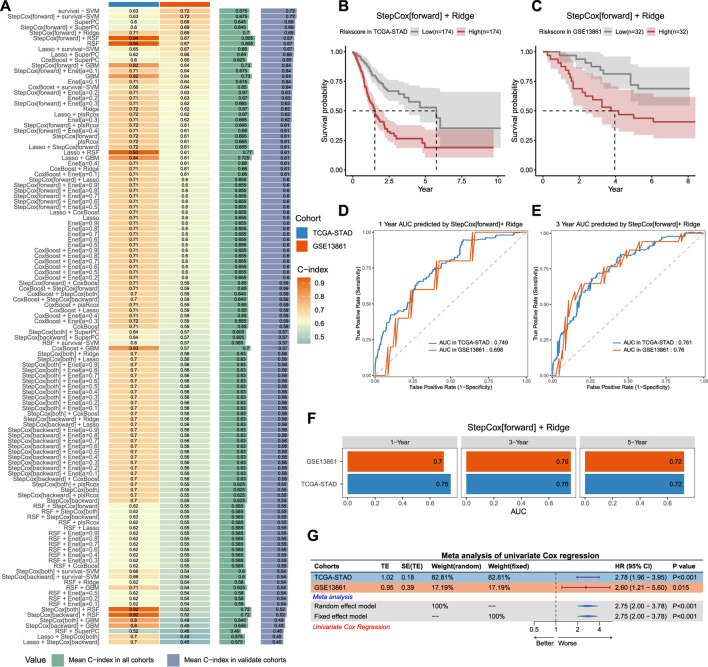
Construction and validation of the mitochondrial-dependent prognostic model in GC. **(A)** Workflow of model construction and performance comparison among 101 combinations of 10 machine learning algorithms using the mime framework. **(B,C)** Kaplan–Meier survival curves comparing overall survival between high- and low-risk groups in training cohorts (TCGA-STAD) and validation cohorts (GSE13861). **(D,E)** Time-dependent ROC curves evaluating the predictive performance of the model for 1- and 3-year overall survival in training cohorts and validation cohorts, respectively. **(F)** Barplots summarize the 1-, 3-, and 5-year AUC values across training cohorts and validation cohorts. **(G)** Forestplot of meta-analysis results integrating univariate Cox regression outcomes from training cohorts and validation cohorts (pooled HR = 2.75, 95% CI: 2.00–3.78, *p* < 0.001). GC: gastric cancer; AUC: area under the curve; ROC: receiver operating characteristic; HR: hazard ratio; CI: confidence interval.

A total of 101 combinations of 10 machine learning algorithms were evaluated to identify the optimal model. The StepCox [forward]+Ridge combination exhibited the highest predictive accuracy, with a mean C-index of 0.71 across training cohorts (TCGA-STAD, n=379) and 0.69 in a validation cohort (GSE13861, n=65, Figure 6A). When patients from were classified into high- and low-risk groups according to the median risk score derived from the model, those in the high-risk group showed significantly worse OS in both training dataset and validation dataset (Figures 6B, C). The predictive performance of the model was further validated using time-dependent ROC analyses. The AUC values for 1-, 3-, and 5-year survival were 0.75, 0.76, and 0.72 in a training dataset, and 0.70, 0.76, and 0.72 in a validation dataset, respectively (Figures 6D-F), indicating robust and consistent predictive ability. Moreover, meta-analysis of univariate Cox regression in the training cohorts and validation cohorts confirmed the prognostic value of the model, yielding a pooled hazard ratio (HR) of 2.75 (95% CI: 2.00-3.78, *P*<0.001) under the fixed-effect model (Figure 6G). These findings demonstrate that the mitochondrial-dependent prognostic model constructed based on mtGenes exhibits strong stability and predictive performance, providing a reliable tool for prognosis evaluation in GC patients.

To evaluate model robustness and the potential for overfitting, we incorporated calibration curves and decision curve analysis (DCA) for 1-, 3-, and 5-year survival. The calibration plots demonstrated generally good agreement between predicted and observed survival probabilities, although some deviations were noted at later time points, likely reflecting limited sample size and dataset heterogeneity. In terms of discrimination, the model exhibited moderate and stable predictive performance across different time horizons, without evidence of over-optimistic performance in the training set. DCA further showed that the model provides a net clinical benefit within a defined range of threshold probabilities, although this advantage is not consistent across all thresholds. Collectively, these results indicate that the model achieves reasonable robustness and potential clinical utility, with no clear signs of substantial overfitting (Supplementary Figure S7).

### 3.10 SCENIC analysis to infer transcription factor networks associated with mitochondrial gene regulation

**FIGURE 7:**
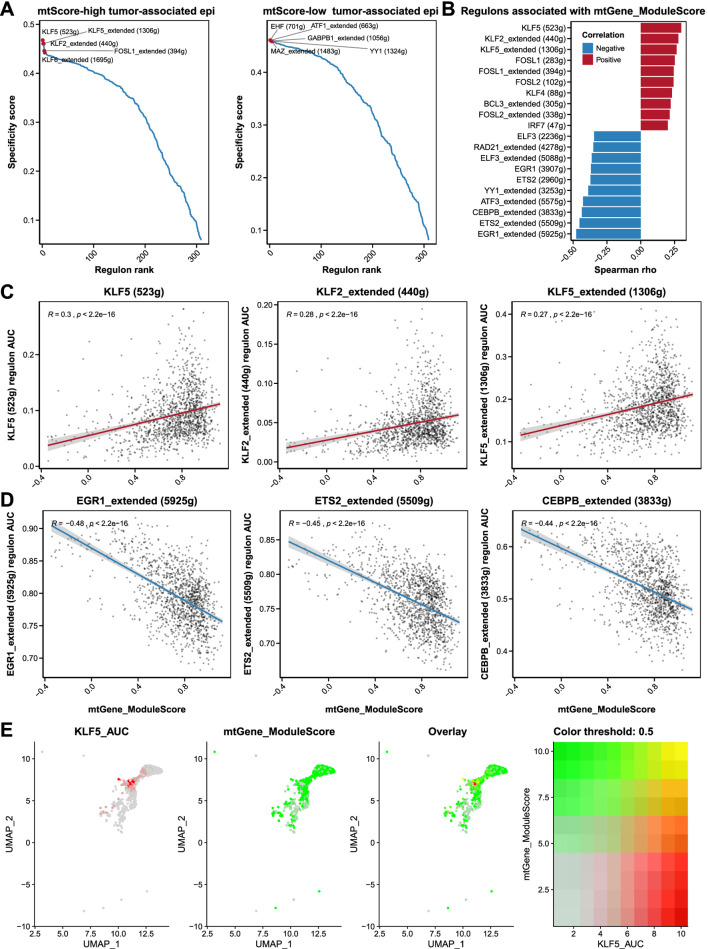
Identification of transcriptional regulators associated with mitochondrial conserved gene score in tumor-associated epithelial cells. **(A)** RSS analysis: ranking of regulons based on specificity scores in mtScore-high and mtScore-low tumor-associated epithelial cells; top-ranked regulons are labeled. **(B)** Bar plot showing Spearman correlation coefficients between regulon activity (AUC) and mtGene_ModuleScore; colors indicate positive and negative correlations. **(C,D)** Scatter plots of top 3 positively and negatively correlated regulons: relationships between regulon activity (AUC) and mtGene_ModuleScore for selected regulons. **(E)** Distribution of KLF5 regulon activity, mtGene_ModuleScore, and their combined visualization in tumor-associated epithelial cells shown by UMAP. RSS: regulon specificity score; AUC: area under the curve; UMAP: uniform manifold approximation and projection.

To further elucidate the regulatory mechanisms underlying mitochondrial homeostasis imbalance, we performed SCENIC analysis to infer transcription factor (TF) regulatory networks associated with mitochondrial gene programs. Regulon specificity score (RSS) analysis identified distinct TF regulatory patterns between mtScore-high and mtScore-low tumor-associated epithelial cells (Figure 7A). Several TFs, including members of the KLF family (KLF5, KLF2), FOSL1, and KLF4, were enriched in mtScore-high cells, whereas TFs such as EGR1, ETS2, and CEBPB were preferentially associated with mtScore-low cells. Correlation analysis further demonstrated that these TFs exhibited consistent positive or negative associations with mtScore at the single-cell level (Figures 7B–D), indicating coordinated transcriptional programs underlying mitochondrial state heterogeneity. UMAP visualization of regulon activity revealed spatial concordance between TF activity and mitochondrial gene scores. For example, KLF5 regulon activity showed co-localization with regions of high mtGene_ModuleScore (Figure 7E), supporting its association with mtScore-high transcriptional states. In contrast, negatively correlated regulons, such as EGR1, displayed distinct spatial distribution patterns relative to mtGene_ModuleScore (Supplementary Figure S8). Together, these findings indicate that mitochondrial homeostasis imbalance in tumor-associated epithelial cells is associated with distinct and spatially organized transcriptional regulatory programs.

In parallel, we applied CellChat analysis to investigate intercellular communication within the tumor microenvironment (Supplementary Figure S9). The results revealed extensive ligand–receptor interactions between tumor-associated epithelial cells and surrounding immune and stromal compartments. In particular, tumor cells exhibited strong outgoing signaling activity toward immune cells, primarily through CXCL and MIF pathways, as well as toward stromal components *via* TGFβ and extracellular matrix (ECM)-related signaling pathways.

## 4 Discussion

Mitochondria participate in cellular energy metabolism and regulate apoptosis, redox balance, and immune signaling (Andrieux et al., 2021). It plays a significant role in GC and holds a potential therapeutic value for the targeting of mitochondria in GC treatment (Tanprasert et al., 2022; Ghosh et al., 2020). However, given the conserved characteristics of mitochondria across species, the expression homeostasis of mitochondrial conserved genes has often been ignored in cancer studies (Wang et al., 2014). In this work, we proposed a hypothetical conceptual framework of “mitochondrial conserved gene expression homeostasis imbalance,” positing that, although mitochondrial gene programs are broadly conserved across tissues, their coordinated expression becomes disrupted in malignancy in a cell-type-resolved manner. Leveraging single-cell transcriptomic analyses, we demonstrate that mitochondrial conserved gene expression is not uniform but instead exhibits distinct, cell-type–specific patterns. Notably, these patterns are significantly reprogrammed between tumor-derived and matched normal cells within the same lineage, underscoring a previously unappreciated layer of mitochondrial dysregulation in cancer.

The expression differences of mitochondrial conserved genes at the cellular subpopulation level are probably overlapped by bulk sequencing. We conducted pesudobluk to compare the mitochondrial conserved gene expression between tumor-derived and normal-derived cells at the single-cell resolution. As we assumed, the expression of 13 mitochondrial conserved genes respectively and the mitochondrial conserved genes score both showed no significant difference between tumor and paired normal tissues (*P*>0.05), while the expression of most mitochondrial conserved genes and the mitochondrial conserved genes score in specific-cell-subtype displayed significant difference between tumor-derived and paired normal-derived tissues (*P*<0.05). Then we summarized the mitochondrial conserved gene expression patterns of different cell types in GC and found that epithelial compartments in GC showed relatively increased expression of mitochondrial conserved genes, while non-epithelial compartments showed relatively decreased expression, indicating a possible resource redistribution or metabolic competition within the tumor microenvironment (Pavlova and Thompson, 2016). This finding may relate to metabolic reprogramming and intercellular mitochondrial dynamics, by which tumor epithelial cells could increase mitochondrial activity to support proliferation (Sahinbegovic et al., 2020), while surrounding cells are potentially suppressed or deprived (Liang et al., 2022). In addition, the suppression of immune cells would impair the anti-tumor competence (Dunn et al., 2004), promote tumor cells proliferation and establish a stable positive feedback. Meanwhile, there are spatial transcriptomics analyses found that “tumor-normal interface” region where tumor cells contact adjacent tissues are characterized with distinct immunometabolic alterations in gastrointestinal tumors (Sun et al., 2023; Wu et al., 2022). A recent research about mitochondria transfer also supports our findings, which revealed that the imbalance of mitochondrial homeostasis may further contribute to immune escape and microenvironment remodeling (Ikeda et al., 2025). Unlike previous studies that focused on isolated mitochondrial alterations, our work establishes a comprehensive hypothesis of “mitochondrial homeostasis imbalance”, considering compartment-associated differences in mtGene expression between epithelial and non-epithelial cells. This hypothetical framework bridges metabolic and immunological observations, as well as provides new insights into mitochondrial coordination across cell populations in GC. Our findings suggest the potential therapeutic value of targeting mitochondria homeostasis in GC treatment, by which may provide new alternative treatment for GC patients in the future. For example, transplanting the mitochondria from normal gastric epithelial cells into GC cells can inhibit the stemness and chemoresistance of the cancer cells, and also suppress tumor growth (Tsai et al., 2025), which reveals that therapies targeting oxidative metabolism or even mitochondrial transplantation strategies may hold promise in restoring homeostasis and improving outcomes in GC.

To identify potential biomarkers reflecting mitochondrial conserved gene homeostasis imbalance and patient prognosis, we found *KRT7* as a poor prognostic marker and *KLRC1* as a favorable prognostic marker in GC. KRT7, as a cytoskeletal protein, is abnormally highly expressed in various cancer tissues and has been proven to promote tumor proliferation and migration (breast cancer, colorectal cancer, pancreatic cancer, etc.) (Chen et al., 2021; Chen et al., 2020; Xu et al., 2024). Studies have shown that high expression of KRT7 is associated with OXPHOS (Hosseinalizadeh et al., 2023), and OXPHOS is an important metabolic pathway for tumor cells to maintain energy balance under stress conditions. *KLRC1* is an immune checkpoint molecule present on the surface of NK cells and CD8^+^ T cells. It mainly binds to the HLA-E molecule on the surface of antigen-presenting cells and transmits inhibitory signals, thereby preventing the immune system from over-activation (Zhang et al., 2015). High expression of KLRC1 indicates that the functions of NK cells and T cells are suppressed, and anti-KLRC1 immunotherapy can enhance anti-tumor immunity by activating T cells and NK cells (Andre et al., 2018; L et al., 2024; van Hall et al., 2019). However, our study observed that it is associated with a better prognosis, which is a seemingly contradictory phenomenon. *KLRC1* itself does not directly participate in metabolic pathways, but its expression and function are closely related to the metabolic state of immune cells. KLRC1 can act as an inhibitory checkpoint receptor contributing to immune exhaustion, particularly in cytotoxic lymphocytes, while also reflecting adaptive immune remodeling within the tumor microenvironment. This dual role may explain the observed associations in our dataset, in which *KLRC1* expression correlates with mitochondrial states and immune-related signaling patterns. In addition, KLRC1 may serve as a marker of immune suppression as well as part of a broader compensatory immune activation/exhaustion axis in gastric cancer, emphasizing the importance of cellular context when interpreting its biological significance. Activated immune cells (such as T cells and NK cells) rely on OXPHOS and glycolysis to provide energy (Steinert et al., 2025). Studies have shown that immune cells with intact mitochondrial function are more likely to rely on OXPHOS to maintain long-term anti-tumor activity (Frisch et al., 2025). In the GC microenvironment, the high expression of *KLRC1* may indirectly protect the mitochondrial function of NK cells and T cells by maintaining their metabolic adaptability, thereby avoiding metabolic failure under continuous antigen stimulation. Detecting the expression of *KRT7* and *KLRC1* could predict the prognosis and help us understand the mitochondrial conserved gene expression homeostasis in the tumor microenvironment, which can offer more personalized guidance for clinical diagnosis and treatment for GC patients. However, the relationship between *KLRC1* and GC and the possible mechanisms remain to be further investigated. Additionally, validation of a large clinical samples will further supports our hypothesis, and our attention should be broadened into more gastrointestinal tumors and even pan-cancers in the future.

## 5 Limitations

This study has several limitations that should be acknowledged. The sample size is relatively limited, particularly at the single-cell level, which may reduce statistical power and limit the generalizability of the findings. GC is highly heterogeneous, and although we applied stratified and integrative analyses, residual tumor heterogeneity may still influence the observed mitochondrial-related transcriptional patterns. In addition, external validation in independent single-cell and bulk transcriptomic cohorts is lacking, which may limit the robustness and clinical generalizability of the proposed signatures. This study is primarily data-driven and hypothesis-generating, therefore, key findings, including the functional roles of mitochondrial conserved genes and surrogate biomarkers, require further experimental validation using approaches such as immunohistochemistry and functional assays in future work.

## 6 Conclusion

This study reveals a significant cell-type-specific imbalance in mitochondrial conserved gene expression in GC and proposes the “mitochondrial conserved gene expression homeostasis imbalance” hypothetical framework. While bulk analysis masked differences, epithelial compartments exhibit relatively elevated mitochondrial gene expression, likely supporting tumor growth. Conversely, non-epithelial compartments show relatively reduced expression, potentially impairing anti-tumor ability and remodeling the microenvironment. These findings offer new insights for targeting mitochondrial homeostasis in GC therapy.

## Data Availability

The original contributions presented in the study are included in the article/Supplementary Material, further inquiries can be directed to the corresponding authors.

## References

[B1] Andre P. Denis C. Soulas C. Bourbon-Caillet C. Lopez J. Arnoux T. (2018). Anti-NKG2A mAb is a checkpoint inhibitor that promotes anti-tumor immunity by unleashing both T and NK cells. Cell 175 (7), 1731–43 e13. 10.1016/j.cell.2018.10.014 30503213 PMC6292840

[B2] Andrieux P. Chevillard C. Cunha-Neto E. Nunes J. P. S. (2021). Mitochondria as a cellular hub in infection and inflammation. Int. J. Mol. Sci. 22 (21), 11338. 10.3390/ijms222111338 34768767 PMC8583510

[B3] Barshad G. Marom S. Cohen T. Mishmar D. (2018). Mitochondrial DNA transcription and its regulation: an evolutionary perspective. Trends Genet. 34 (9), 682. 10.1016/j.tig.2018.05.009 29945721

[B4] Bray F. Laversanne M. Sung H. Ferlay J. Siegel R. L. Soerjomataram I. (2024). Global cancer statistics 2022: GLOBOCAN estimates of incidence and mortality worldwide for 36 cancers in 185 countries. CA Cancer J. Clin. 74 (3), 229. 10.3322/caac.21834 38572751

[B5] Burr S. P. Klimm F. Glynos A. Prater M. Sendon P. Nash P. (2023). Cell lineage-specific mitochondrial resilience during mammalian organogenesis. Cell 186 (6), 1212–2921. 10.1016/j.cell.2023.01.034 36827974

[B6] Chen S. Su T. Zhang Y. Lee A. He J. Ge Q. (2020). Fusobacterium nucleatum promotes colorectal cancer metastasis by modulating KRT7-AS/KRT7. Gut Microbes 11 (3), 511–625. 10.1080/19490976.2019.1695494 31910722 PMC7524269

[B7] Chen F. Chen Z. Guan T. Zhou Y. Ge L. Zhang H. (2021). N(6) -Methyladenosine regulates mRNA stability and translation efficiency of KRT7 to promote breast cancer lung metastasis. Cancer Res. 81 (11), 2847. 10.1158/0008-5472.CAN-20-3779 33795252

[B8] Chen F. Xue Y. Zhang W. Zhou H. Zhou Z. Chen T. (2024). The role of mitochondria in tumor metastasis and advances in mitochondria-targeted cancer therapy. Cancer Metastasis Rev. 43 (4), 1419. 10.1007/s10555-024-10211-9 39307891 PMC11554835

[B9] Dunn G. P. Old L. J. Schreiber R. D. (2004). The immunobiology of cancer immunosurveillance and immunoediting. Immunity 21 (2), 137. 10.1016/j.immuni.2004.07.017 15308095

[B10] Frisch A. T. Wang Y. Xie B. Yang A. Ford B. R. Joshi S. (2025). Redirecting glucose flux during in vitro expansion generates epigenetically and metabolically superior T cells for cancer immunotherapy. Cell Metab. 37 (4), 870–888. 10.1016/j.cmet.2024.12.007 39879981 PMC12101091

[B11] Gene Ontology Consortium (2015). Gene Ontology Consortium: going forward. Nucleic Acids Res. (43), D1049–D1056. 10.1093/nar/gku1179 25428369 PMC4383973

[B12] Ghosh P. Vidal C. Dey S. Zhang L. (2020). Mitochondria targeting as an effective strategy for cancer therapy. Int. J. Mol. Sci. 21 (9), 3363. 10.3390/ijms21093363 32397535 PMC7247703

[B13] Guan W. L. He Y. Xu R. H. (2023). Gastric cancer treatment: recent progress and future perspectives. J. Hematol. Oncol. 16 (1), 57. 10.1186/s13045-023-01451-3 37245017 PMC10225110

[B14] Hanzelmann S. Castelo R. Guinney J. (2013). GSVA: gene set variation analysis for microarray and RNA-seq data. BMC Bioinformatics 14 (7), 7. 10.1186/1471-2105-14-7 23323831 PMC3618321

[B15] Hao Y. Hao S. Andersen-Nissen E. Mauck W. M. 3rd Zheng S. Butler A. (2021). Integrated analysis of multimodal single-cell data. Cell 184 (13), 3573. 10.1016/j.cell.2021.04.048 34062119 PMC8238499

[B16] Harrington J. S. Ryter S. W. Plataki M. Price D. R. Choi A. M. K. (2023). Mitochondria in health, disease, and aging. Physiol. Rev. 103 (4), 2349. 10.1152/physrev.00058.2021 37021870 PMC10393386

[B17] Hosseinalizadeh H. Hussain Q. M. Poshtchaman Z. Ahsan M. Amin A. H. Naghavi S. (2023). Emerging insights into keratin 7 roles in tumor progression and metastasis of cancers. Front. Oncol. 13, 1243871. 10.3389/fonc.2023.1243871 38260844 PMC10800941

[B18] Ikeda H. Kawase K. Nishi T. Watanabe T. Takenaga K. Inozume T. (2025). Immune evasion through mitochondrial transfer in the tumour microenvironment. Nature 638 (8049), 225. 10.1038/s41586-024-08439-0 39843734 PMC11798832

[B19] Kuo C. L. Chou H. Y. Chiu Y. C. Cheng A. N. Fan C. C. Chang Y. N. (2020). Mitochondrial oxidative stress by Lon-PYCR1 maintains an immunosuppressive tumor microenvironment that promotes cancer progression and metastasis. Cancer Lett. 474, 138. 10.1016/j.canlet.2020.01.019 31987921

[B20] Li Y. Li Z. Tang Y. Zhuang X. Feng W. Boor P. P. C. (2024). Unlocking the therapeutic potential of the NKG2A-HLA-E immune checkpoint pathway in T cells and NK cells for cancer immunotherapy. J. Immunother. Cancer 12 (10), e009934. 10.1136/jitc-2024-009934 39486805 PMC11529472

[B21] Liang L. Li W. Li X. Jin X. Liao Q. Li Y. (2022). Reverse Warburg effect of cancer-associated fibroblasts. Int. J. Oncol. 60(6), 67. Available online at: https://www.ncbi.nlm.nih.gov/pubmed/35425996/. 10.3892/ijo.2022.5357 35425996

[B22] Liu Y. Zhang Z. Wang J. Chen C. Tang X. Zhu J. (2019). Metabolic reprogramming results in abnormal glycolysis in gastric cancer: a review. Onco Targets Ther. 12, 1195. 10.2147/OTT.S189687 30863087 PMC6389007

[B23] Liu H. Zhang W. Zhang Y. Adegboro A. A. Fasoranti D. O. Dai L. (2024). Mime: a flexible machine-learning framework to construct and visualize models for clinical characteristics prediction and feature selection. Comput. Struct. Biotechnol. J. 23, 2798. 10.1016/j.csbj.2024.06.035 39055398 PMC11269309

[B24] Nitsch L. Lareau C. A. Ludwig L. S. (2024). Mitochondrial genetics through the lens of single-cell multi-omics. Nat. Genet. 56 (7), 1355. 10.1038/s41588-024-01794-8 38951641 PMC11260401

[B25] Pavlova N. N. Thompson C. B. (2016). The emerging hallmarks of cancer metabolism. Cell Metab. 23 (1), 27–47. 10.1016/j.cmet.2015.12.006 26771115 PMC4715268

[B26] Sahinbegovic H. Jelinek T. Hrdinka M. Bago J. R. Turi M. Sevcikova T. (2020). Intercellular mitochondrial transfer in the tumor microenvironment. Cancers (Basel) 12 (7), 1787. 10.3390/cancers12071787 32635428 PMC7407231

[B27] Scarpulla R. C. (2008). Transcriptional paradigms in mammalian mitochondrial biogenesis and function. Physiol. Rev. 88 (2), 611. 10.1152/physrev.00025.2007 18391175

[B28] Shtolz N. Mishmar D. (2023). The metazoan landscape of mitochondrial DNA gene order and content is shaped by selection and affects mitochondrial transcription. Commun. Biol. 6 (1), 93. 10.1038/s42003-023-04471-4 36690686 PMC9871016

[B29] Smyth E. C. Nilsson M. Grabsch H. I. van Grieken N. C. Lordick F. (2020). Gastric cancer. Lancet 396 (10251), 635. 10.1016/S0140-6736(20)31288-5 32861308

[B30] Steinert E. M. Furtado Bruza B. Danchine V. D. Grant R. A. Vasan K. Kharel A. (2025). Mitochondrial respiration is necessary for CD8(+) T cell proliferation and cell fate. Nat. Immunol. 26 (8), 1267. 10.1038/s41590-025-02202-x 40670617 PMC12307223

[B31] Sturm G. Karan K. R. Monzel A. S. Santhanam B. Taivassalo T. Bris C. (2023). OxPhos defects cause hypermetabolism and reduce lifespan in cells and in patients with mitochondrial diseases. Commun. Biol. 6 (1), 22. 10.1038/s42003-022-04303-x 36635485 PMC9837150

[B32] Sun C. Wang A. Zhou Y. Chen P. Wang X. Huang J. (2023). Spatially resolved multi-omics highlights cell-specific metabolic remodeling and interactions in gastric cancer. Nat. Commun. 14 (1), 2692. 10.1038/s41467-023-38360-5 37164975 PMC10172194

[B33] Tanprasert P. Limpakan Yamada S. Chattipakorn S. C. Chattipakorn N. Shinlapawittayatorn K. (2022). Targeting mitochondria as a therapeutic anti-gastric cancer approach. Apoptosis 27 (3-4), 163. 10.1007/s10495-022-01709-0 35089473

[B34] Tsai H. Y. Tsai K. J. Wu D. C. Huang Y. B. Lin M. W. (2025). Transplantation of gastric epithelial mitochondria into human gastric cancer cells inhibits tumor growth and enhances chemosensitivity by reducing cancer stemness and modulating gastric cancer metabolism. Stem Cell Res. Ther. 16 (1), 87. 10.1186/s13287-025-04223-7 39988680 PMC11849191

[B35] van Hall T. Andre P. Horowitz A. Ruan D. F. Borst L. Zerbib R. (2019). Monalizumab: inhibiting the novel immune checkpoint NKG2A. J. Immunother. Cancer 7 (1), 263. 10.1186/s40425-019-0761-3 31623687 PMC6798508

[B36] Wang G. Yang E. Mandhan I. Brinkmeyer-Langford C. L. Cai J. J. (2014). Population-level expression variability of mitochondrial DNA-encoded genes in humans. Eur. J. Hum. Genet. 22 (9), 1093. 10.1038/ejhg.2013.293 24398800 PMC4135407

[B37] Wang R. Song S. Qin J. Yoshimura K. Peng F. Chu Y. (2023). Evolution of immune and stromal cell states and ecotypes during gastric adenocarcinoma progression. Cancer Cell 41 (8), 1407. 10.1016/j.ccell.2023.06.005 37419119 PMC10528152

[B38] Wroblewski L. E. Peek R. M. Jr. (2010). Wilson K T Helicobacter pylori and gastric cancer: factors that modulate disease risk. Clin. Microbiol. Rev. 23 (4), 713. 10.1128/CMR.00011-10 20930071 PMC2952980

[B39] Wu Y. Yang S. Ma J. Chen Z. Song G. Rao D. (2022). Spatiotemporal immune landscape of colorectal cancer liver metastasis at single-cell level. Cancer Discov. 12 (1), 134. 10.1158/2159-8290.CD-21-0316 34417225

[B40] Xu C. Wang S. Sun Y. (2024). The role of KRT7 in metastasis and prognosis of pancreatic cancer. Cancer Cell Int. 24 (1), 321. 10.1186/s12935-024-03500-4 39300449 PMC11412054

[B41] Yu G. Wang L. G. Han Y. He Q. Y. (2012). clusterProfiler: an R package for comparing biological themes among gene clusters. OMICS 16 (5), 284. 10.1089/omi.2011.0118 22455463 PMC3339379

[B42] Zhang X. Lan Y. Xu J. Quan F. Zhao E. Deng C. (2019). CellMarker: a manually curated resource of cell markers in human and mouse. Nucleic Acids Res. 47 (D1), D721. 10.1093/nar/gky900 30289549 PMC6323899

[B43] Zhang J. Basher F. Wu J. D. (2015). NKG2D ligands in tumor immunity: two sides of a coin. Front. Immunol. 6, 97. 10.3389/fimmu.2015.00097 25788898 PMC4349182

[B44] Zhang L. Zhang W. Li Z. Lin S. Zheng T. Hao B. (2022). Mitochondria dysfunction in CD8+ T cells as an important contributing factor for cancer development and a potential target for cancer treatment: a review. J. Exp. Clin. Cancer Res. 41 (1), 227. 10.1186/s13046-022-02439-6 35864520 PMC9306053

[B45] Zhang L. Wei Y. Yuan S. Sun L. (2023). Targeting mitochondrial metabolic reprogramming as a potential approach for cancer therapy. Int. J. Mol. Sci. 24 (5), 4954. 10.3390/ijms24054954 36902385 PMC10003438

[B46] Zhao L. Liu Y. Zhang S. Wei L. Cheng H. Wang J. (2022). Impacts and mechanisms of metabolic reprogramming of tumor microenvironment for immunotherapy in gastric cancer. Cell Death Dis. 13 (4), 378. 10.1038/s41419-022-04821-w 35444235 PMC9021207

