# Supplementary figures and images for: Single-cell and pseudobulk analyses reveal hidden mitochondrial expression imbalance in gastric cancer
Source: Front Genet. 2026 Jun 17;17:1826214. doi: 10.3389/fgene.2026.1826214 (PMC13318257; doi:10.3389/fgene.2026.1826214)

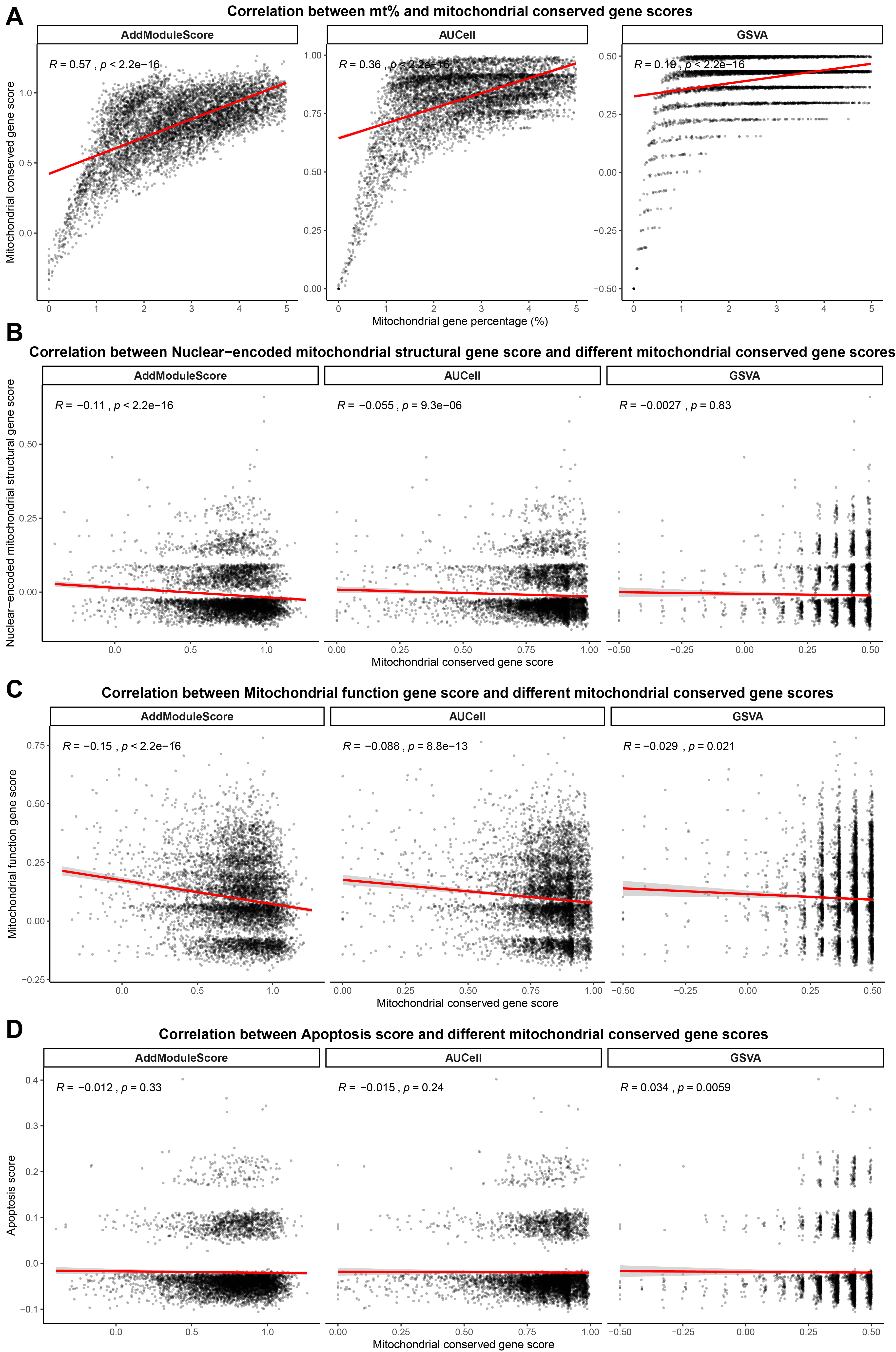

Supplement: Supplementary file 1 [file Image3.jpeg]

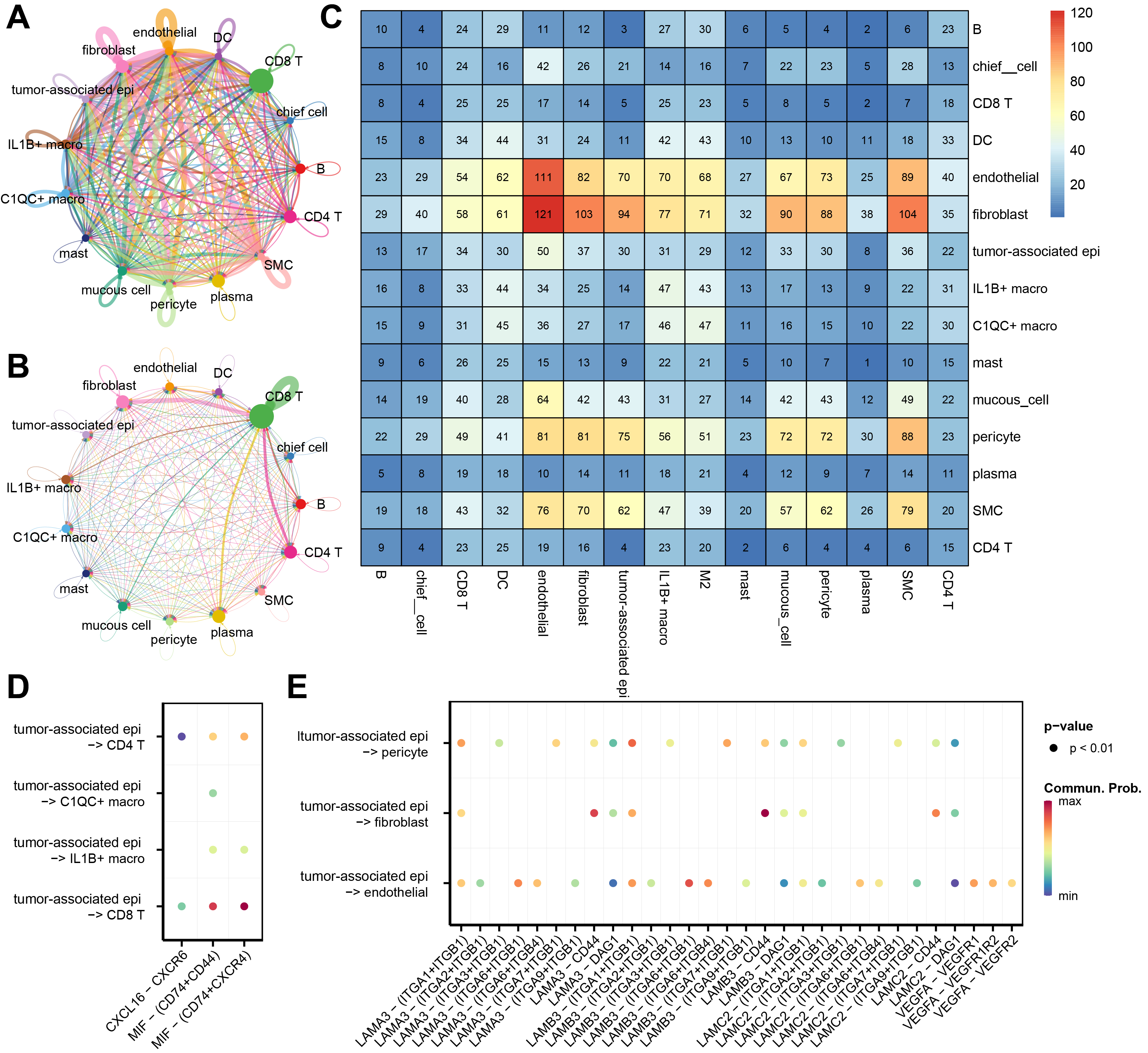

Supplement: Supplementary file 2 [file Image9.jpeg]

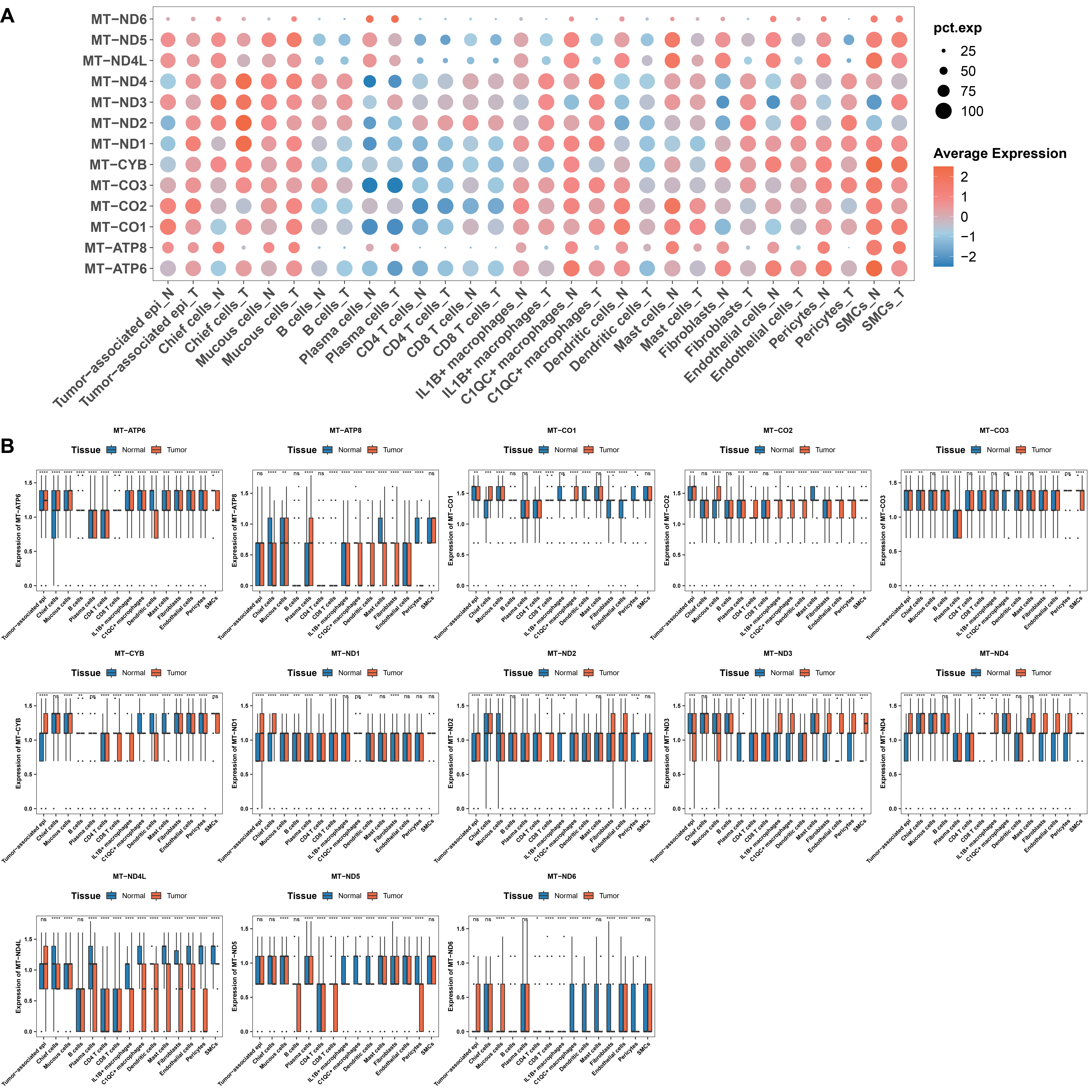

Supplement: Supplementary file 4 [file Image4.jpeg]

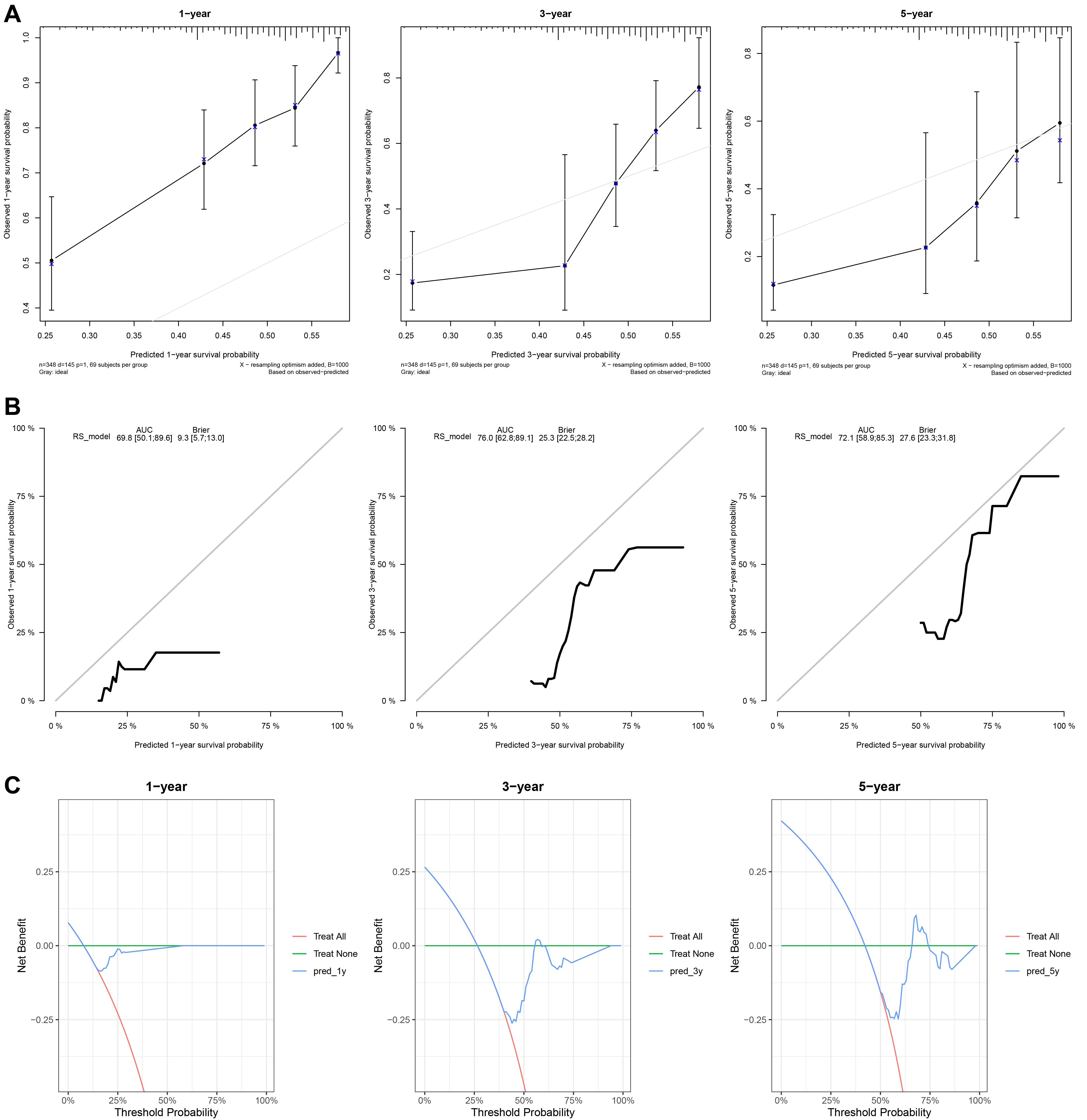

Supplement: Supplementary file 5 [file Image7.jpeg]

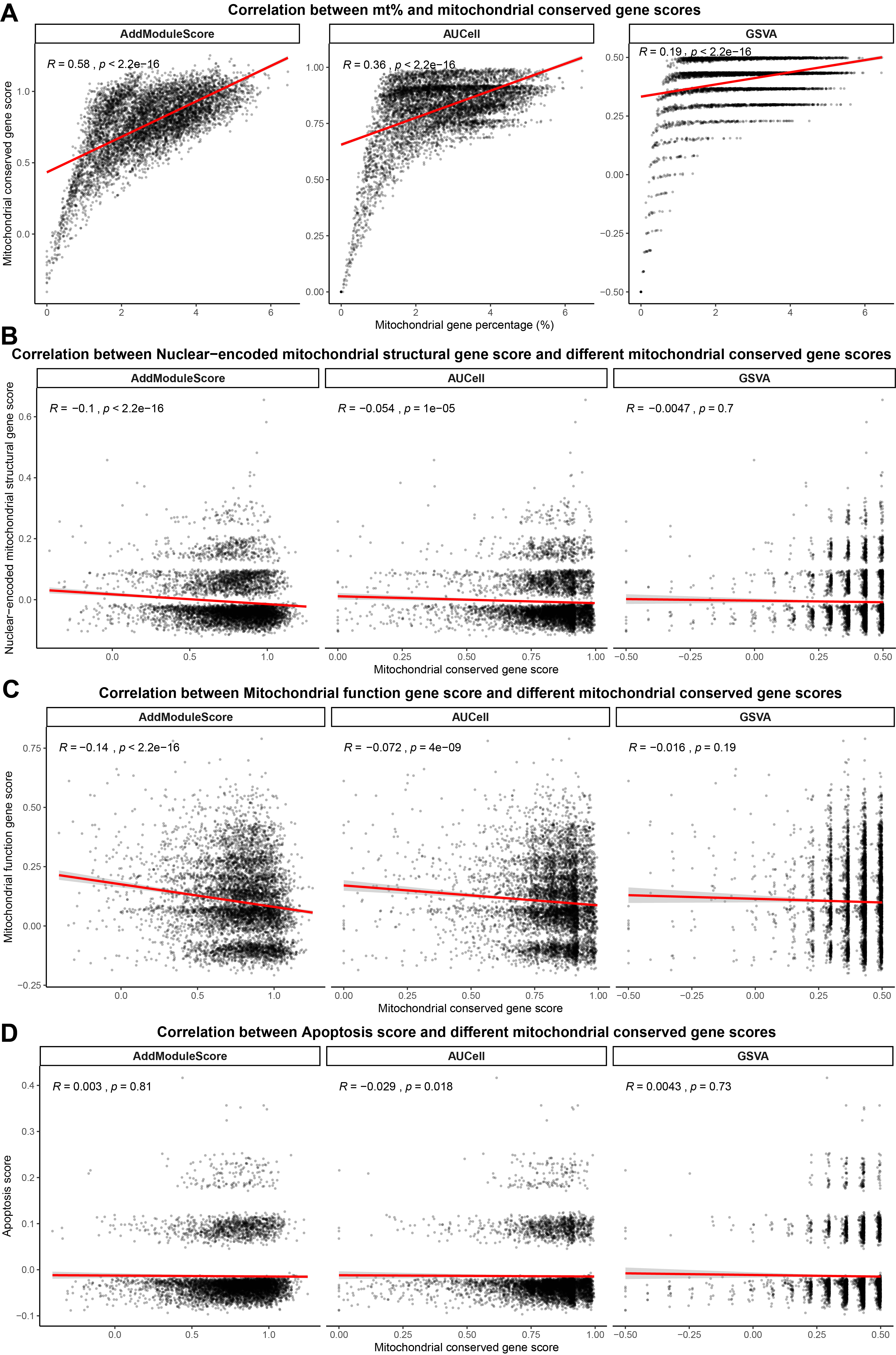

Supplement: Supplementary file 6 [file Image2.jpeg]

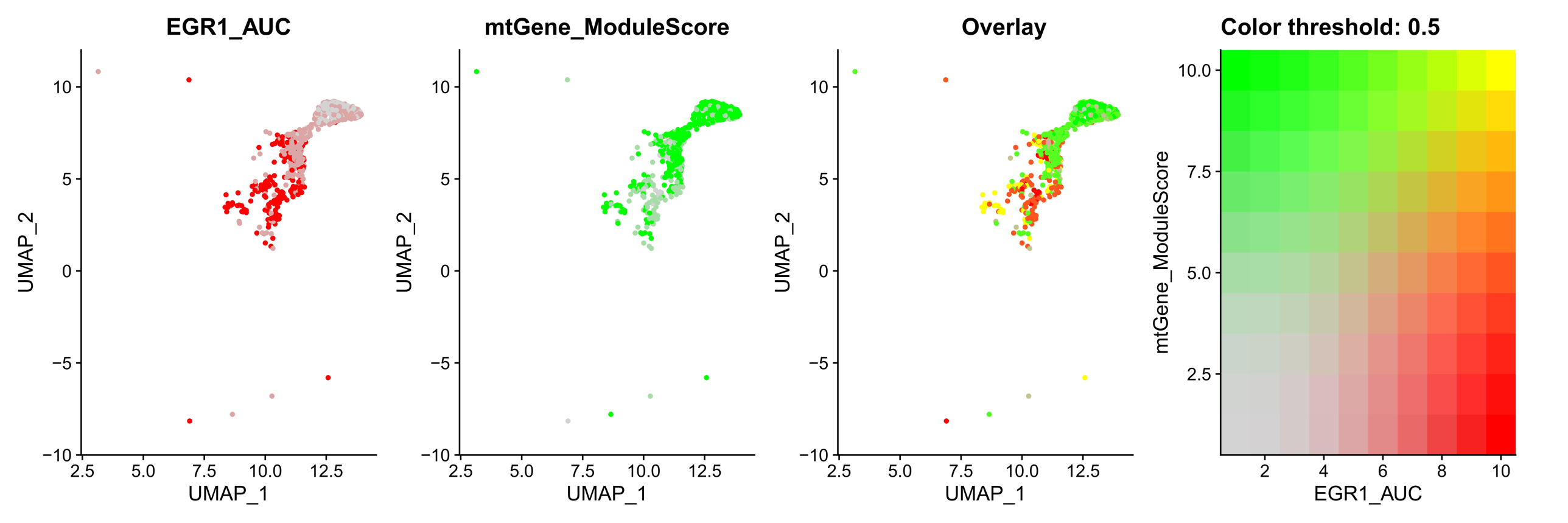

Supplement: Supplementary file 9 [file Image8.jpeg]

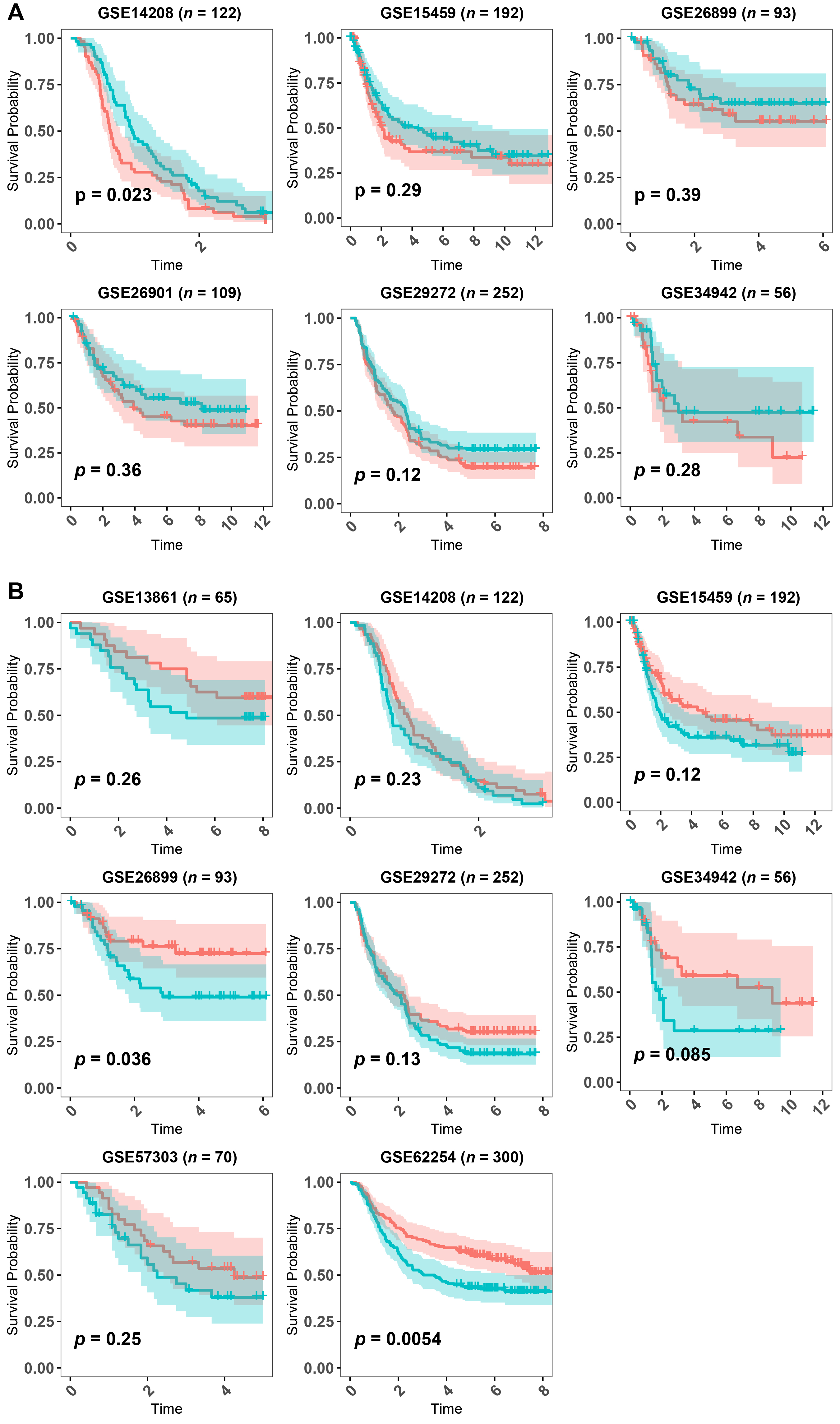

Supplement: Supplementary file 10 [file Image6.jpeg]
